# Differentiated-Type Intraepithelial Neoplasia-Like Lesion Associated with Squamous Cell Carcinoma of the Anus: A Case Report with Molecular Profile

**DOI:** 10.1155/2019/2301640

**Published:** 2019-01-27

**Authors:** Caroline Koopmansch, Calliope Maris, Pieter Demetter, Jean Van de Stadt, Alain Hendlisz, Nicky D'Haene, Jean-Christophe Noël

**Affiliations:** ^1^Department of Pathology, Erasme University Hospital, Free University of Brussels (ULB), Brussels, Belgium; ^2^Department of Pathology, Jules Bordet Institute, Free University of Brussels (ULB), Brussels, Belgium; ^3^Department of Digestive Surgery, Erasme University Hospital, Free University of Brussels (ULB), Brussels, Belgium; ^4^Digestive Oncology Unit, Medicine Department, Jules Bordet Institute, Free University of Brussels (ULB), Brussels, Belgium

## Abstract

Differentiated-type Intraepithelial Neoplasia (DIN) is defined as HPV-negative squamous intraepithelial proliferation with abnormal keratinocyte differentiation and basal cell atypia, originally described in the vulva, with following descriptions in the oral cavity. DIN occurring in the anus is quite rare, and to the best of our knowledge, only one publication reported it. In this report, we describe the clinicopathological features of this entity on anal margin, associated with invasive squamous cell carcinoma. In addition, using the next generation sequencing (NGS) technique, we have demonstrated TP53 mutation in the invasive component but not in the associated DIN-like lesion, where p53 immunohistochemical expression was restricted to basal layers.

## 1. Introduction

Differentiated-type Intraepithelial Neoplasia (DIN) is defined as HPV-negative squamous intraepithelial proliferation with abnormal keratinocyte differentiation and basal cell atypia [1]. This pathological entity was originally described in the vulva, with the following descriptions in the oral cavity [2–5] and the genitourinary tract, especially the penis [6–8]. In the vulva, this lesion is associated with lichen sclerosus or planus and often associated with keratinizing squamous cell carcinoma (SCC).

To the best of our knowledge, only one publication reported DIN in the anus [9]. Terminology concerning this lesion is confusing as it is not described in the* WHO Classification of Tumours of the Digestive System*. Indeed, precursor lesions reported in the anal canal are “classical” anal intraepithelial neoplasia, mostly HPV-related [10]. The prevalence of HPV infection in anal carcinoma (84.3%) approaches that reported in cervical carcinoma (87.3%), for which HPV infection is considered a necessary cause. HPV prevalence is much lower in vulvar carcinoma (40.4%) [11].

In the vulva, DIN is classically associated with* TP53* mutations and will be p53 immunopositive when missense mutations are present. Some cases shared identical* TP53* mutations in both DIN and SCC [12].

Therefore, the aim of the present study is to assess the molecular profile of this entity in the anus using the next generation sequencing (NGS) technique in correlation with immunohistochemical data.

## 2. Case Presentation

**Figure 1:**
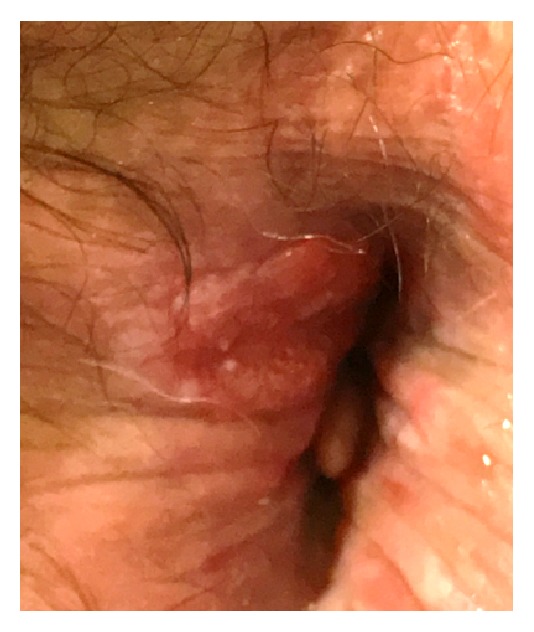
Clinical aspect of the lesion of the anal margin.

A 59-year-old man presented in December 2017 an indurated lesion of the anal margin causing burning sensation, measuring 1 cm (Figure 1).

**Figure 2:**
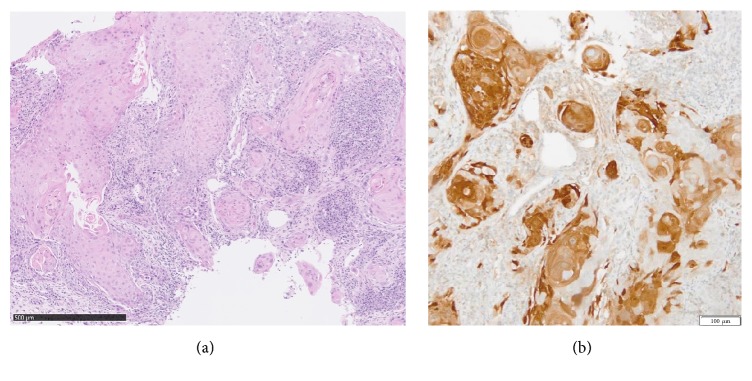
Microscopic aspect on biopsy, revealing moderately differentiated squamous cell carcinoma (a), with irregular/heterogenous positivity for p16 immunohistochemistry (b).

The biopsy revealed moderately differentiated squamous cell carcinoma. Using immunohistochemistry, irregular/heterogenous positivity for p16 protein was observed (Figure 2).

The detection of High Risk-HPV DNA (HPV 16, 18, 31, 33, 35, 39, 45, 51, 52, 56, 59, 66, and 68) from the paraffin-embedded sample using the BD onclarity HPV assay (BD diagnostics, Sparks, USA) was negative [13].

The tumor was classified cT1 and treated by radiotherapy until February 2018. In May 2018, after a period of complete response, the patient noted the reappearance of an indurated and painful area near the anal margin. The patient underwent excision in June 2018.

Macroscopically, an irregular and ulcerated lesion occupying the near totality of a mucous ellipse measuring 26x15 mm was observed. This lesion was covered by a white coat.

**Figure 3:**
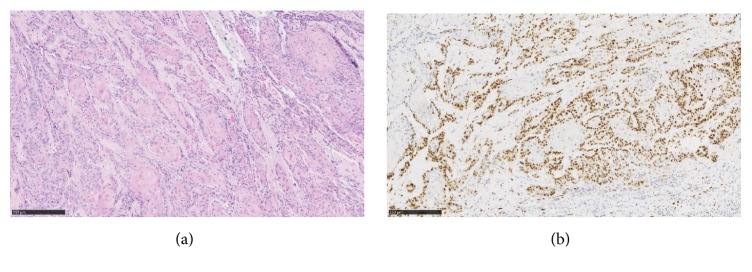
Microscopic and immunohistochemical aspects of 1st excision. Tumor consisted of nests of invasive squamous cell carcinoma, with focal keratinization (a). p53 appeared continuous and limited to the periphery of invasive nests, with strong intensity (b).

Microscopically, the tumor consisted of nests of invasive squamous cell carcinoma, moderately differentiated. Lateral margins were positive. Using immunohistochemistry, tumor was negative for p16 (clone* ink4a E6H4, ready to use, Roche*). Immunoreactivity of p53 (clone* DO-7, 1:200, Dako Agilent*) appeared continuous and limited to the periphery of invasive nests, with strong intensity (Figure 3). The tumor was classified rpT2Nx.

Gene mutation testing was performed by NGS, as we have previously described [14, 15], with a panel of 50 genes described in Table 1. One mutation was found: G279fs*∗*4 (c.833.834insGAGTCGAAACTCCACGCACAAACACGGACAGGAC) frameshift mutation of the* TP53* gene.

In addition, the detection of High Risk-HPV DNA was negative [13].

Due to the positive margins and the classification, PET-CT was realized and was negative. Complementary resection was performed in July 2018.

**Figure 4:**
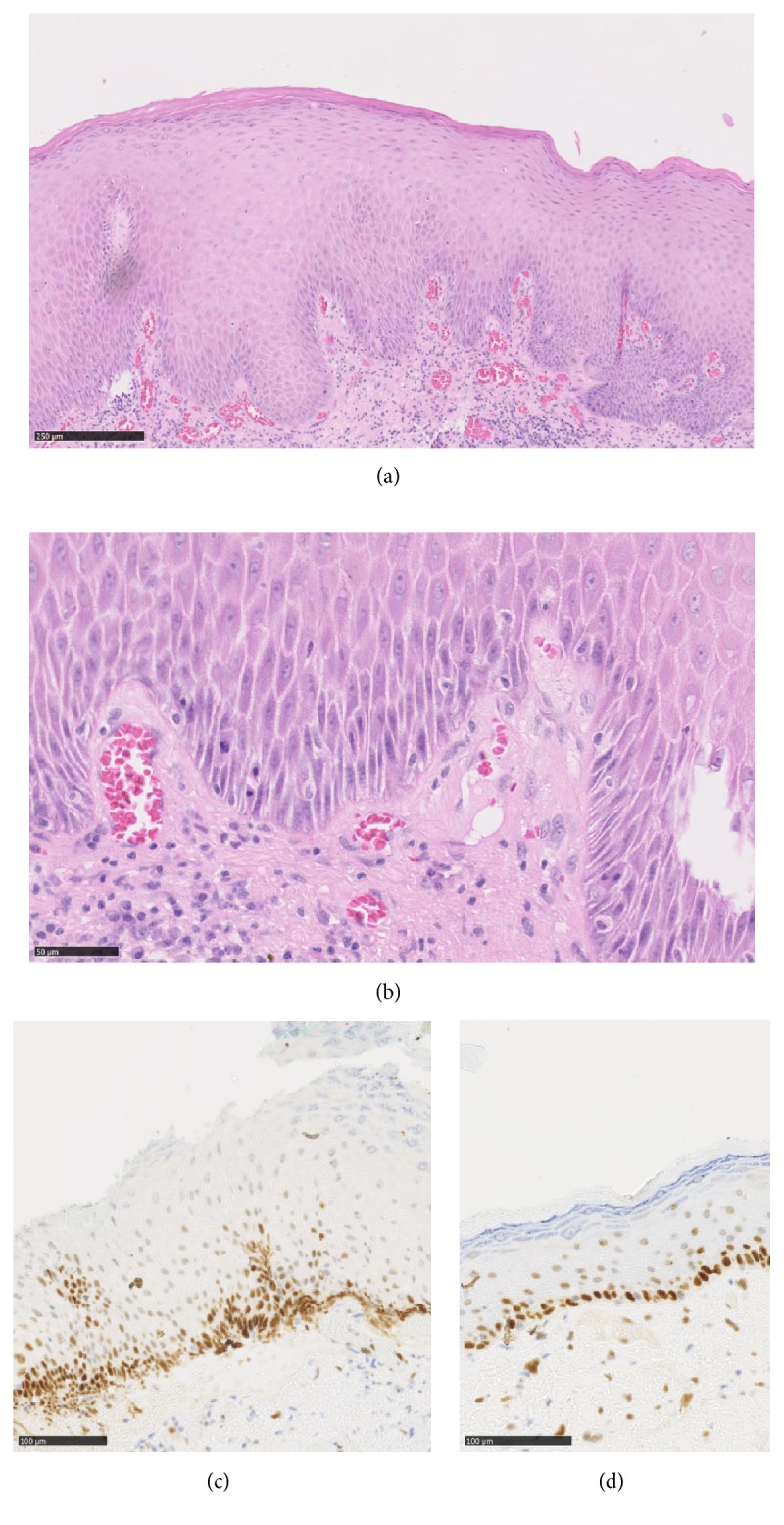
Microscopic and immunohistochemical aspects of 2nd excision. At low power view, the epithelium appeared thickened with parakeratosis and elongated rete ridges (a). At high power view, note the disorderly basal cell layer, prominent intercellular bridges, prominent nucleoli, and mitosis (b). Immunoreactivity of p53 appeared limited to the basal and suprabasal layers of the epithelium, with weak to moderate intensity (c). Immunoreactivity of p53 in adjacent “normal” epithelium (d).

Macroscopically, 2nd resection showed an ulcerated mucous ellipse.Microscopically, borders of the ulceration revealed a thickened epithelium with parakeratosis, elongated rete ridges, disorderly basal cell layer, prominent intercellular bridges, and mitosis. Using immunohistochemistry, just like in 1st resection, lesion was negative for p16. Immunoreactivity of p53 appeared limited to the basal and suprabasal layers of the epithelium, with weak to moderate intensity (Figure 4).

According to all these pathological data, the diagnosis of differentiated-type intraepithelial neoplasia (DIN) was suggested.

Gene mutation testing was performed in this DIN-like lesion, but no mutation was found.

## 3. Discussion

Tumors of the anus and perianal skin are rare. With standard treatment, complete and durable remission can be achieved in the majority of patients. However, locoregional failure rates vary between 16% and 33%: these patients do not respond to therapy or relapse early after treatment [16], such as the patient presented in this case.

Several studies indicated that HPV-/P16- anal cancers had significant worse overall survival and relapse-free survival, compared to HPV+/P16+ anal cancers [17, 18]. In contrast to HPV+ anal cancers, HPV- anal cancers frequently carry TP53 mutations, suggesting that there might be large difference in the genetics of HPV+ versus HPV- tumors [18]. Moreover, loss of p53 function has been linked to resistance to radiotherapy in head and neck SCC [19].

In the present study, we analyzed for the first time molecular profile of both DIN-like lesion and associated SCC in the anus. Such analysis has already been done in the vulva, showing* TP53* mutations in 6 out of 10 cases of DIN (60%) and in 4 out of 5 DIN-associated SCC (80%) [12]. In the present case,* TP53* frameshift mutation was observed only in the SCC. The frameshift (insertion) mutation of the TP53 gene we observed is not reported in the COSMIC database (cancer.sanger.ac.uk) [20]. Other G279 insertion-frameshift mutations of unknown pathogenic significance were previously reported, in liver, larynx, skin, and bladder carcinomas.* TP53* frameshift mutations in other amino acid positions have been reported in anal carcinoma, without functional consequences and variable associated immunoreactivity of p53 [18].

DIN is a subtle and difficult histopathological diagnosis, with a low interobserver agreement [21].

Histological and immunohistochemical characteristics present overlap with other entities, such as lichen sclerosus, squamous cell hyperplasia, or inflammatory disorders. Increased p53 staining can be seen in 5-61% of lichen sclerosus and up to 40% of squamous cell hyperplasia and is thought to be due to increased oxidative stress. Moreover, some authors suspect that atypical lichen sclerosus, showing increased p53 staining, may represent a very early form of DIN [22].

Therefore, we believe these entities are a spectrum of lesions sharing common histological features, where TP53 mutation could be a further event in anal SCC carcinogenesis.

In conclusion, we described a potential precursor lesion of SCC in the anus analogous to DIN in the oral cavity and vulva. The recognition of such a precursor should lead to a careful analysis of the HPV status and the molecular profile of cancer to detect the presence of TP53 mutations. Furthermore, studies investigating prognostic impact of such mutations in DIN-like lesions and associated SCC in the anus are warranted.

## Figures and Tables

**Table 1:** Cancer panel used by NGS.

ABL1	EGFR	GNAQ	KRAS	PTPN11
AKT1	ERBB2	GNAS	MET	RB1

ALK	ERBB4	HNF1A	MLH1	RET

APC	EZH2	HRAS	MPL	SMAD4

ATM	FBXW7	IDH1	NMP1	SMARCB1

BRAF	FGFR1	IDH2	NOTCH1	SMO

CDH1	FGFR2	JAK2	NRAS	SRC

CDKN2A	FGFR3	JAK3	PDGFRA	STK11

CSF1R	FLT3	KDR	PIK3CA	TP53

CTNNB1	GNA11	KIT	PTEN	VHL
